# Modulation of Bromo- and Extra-Terminal Domain (BET) Proteins Exerts Neuroprotective Effects in Cell Culture Models of Parkinson’s Disease

**DOI:** 10.3390/biomedicines14010244

**Published:** 2026-01-21

**Authors:** Noemi Martella, Daniele Pensabene, Mayra Colardo, Maurizio Muzzi, Emanuele Bisesto, Michela Varone, Giuseppina Caretti, Angela Di Porzio, Valentina Barrella, Arianna Mazzoli, Sabrina Di Bartolomeo, Sandra Moreno, Marco Segatto

**Affiliations:** 1 Department of Biosciences and Territory, University of Molise, Contrada Fonte Lappone, 86090 Pesche, Italy; noemi.martella@unimol.it (N.M.); d.pensabene@studenti.unimol.it (D.P.); mayra.colardo@unimol.it (M.C.); maurizio.muzzi@unimol.it (M.M.); e.bisesto@studenti.unimol.it (E.B.); m.varone@studenti.unimol.it (M.V.); sabrina.dibartolomeo@unimol.it (S.D.B.); 2 Department of Science, University Roma Tre, Viale Marconi 446, 00146 Rome, Italy; sandra.moreno@uniroma3.it; 3 Department of Biosciences, University of Milan, Via Celoria 26, 20133 Milan, Italy; giuseppina.caretti@unimi.it; 4 Department of Biology, University of Naples Federico II, Complesso Universitario Monte Sant’Angelo, Via Cinthia, 80126 Naples, Italy; angela.diporzio@unina.it (A.D.P.); valentina.barrella@unina.it (V.B.); arianna.mazzoli@unina.it (A.M.)

**Keywords:** autophagy, JQ1, mitochondria, neurodegeneration, oxidative stress, Parkinson’s disease

## Abstract

**Background/Objectives**: Parkinson’s disease (PD) is one of the most prevalent neurodegenerative disorders. Despite its multifactorial etiology, PD pathophysiology shared specific features such as cytoplasmic α-synuclein inclusions, oxidative stress, mitochondrial dysfunction, and impaired autophagy. Bromodomain and Extra-Terminal domain (BET) proteins, functioning as epigenetic readers, have recently emerged as promising therapeutic targets due to their regulatory role in redox homeostasis, neuroinflammation, and autophagy. However, their potential involvement in PD pathophysiology remains largely unexplored. Therefore, we aimed at evaluating whether BET modulation could ameliorate the parkinsonian phenotype in two cellular models. **Methods**: Differentiated SH-SY5Y and N1E-115 neuronal cells were exposed to rotenone toxin to mimic PD phenotype and co-treated with the small BET inhibitor JQ1. **Results**: BET inhibition significantly counteracted rotenone-induced cell death, neuromorphological alterations, and α-synuclein accumulation. These protective effects were accompanied by restoration of redox balance, as indicated by enhanced activation of the antioxidant system and suppression of the pro-oxidant NADPH oxidase complex. Moreover, JQ1 treatment alleviated mitochondrial dysfunction and corrected autophagy impairments triggered by rotenone. **Conclusions**: These data highlight a novel role for BET proteins in neurodegeneration, suggesting that their modulation may represent a promising approach to counteract PD neuropathology.

## 1. Introduction

Parkinson’s disease (PD) is the second most common neurodegenerative disorder after Alzheimer’s disease (AD), affecting over 6 million people worldwide with a significant incidence increase in the elder population [1,2,3]. PD is characterized by the progressive loss of motor control, bradykinesia, resting tremors, and muscle rigidity. Such clinical symptoms depend on the gradual dopaminergic cell death in the *Substantia Nigra pars compacta* (SNpc), a brain region critically involved in motor control. Therefore, dopamine loss together with alterations in the basal ganglia circuitry determines the onset of parkinsonism [4,5]. Decades-worth of scientific evidence helped to unveil several etiological events: ~5% of patients display mutations affecting various genes (e.g., *SNCA* and *LRRK2*) associated with the familial form of PD, although most cases are sporadic and dependent on the interaction between genetic susceptibility and environmental risk factors (i.e., exposure to pesticides). Despite the nebulous etiology, familial and sporadic PD share pathophysiological hallmarks, such as α-synuclein cytoplasmic inclusions (Lewy’s bodies), oxidative stress, mitochondrial dysfunction, and autophagy impairment [5,6]. Interestingly, all these pathological aspects are tightly linked and contribute significantly to neuronal death. For instance, the disruption of mitochondrial complex I, a common feature in PD, reduces ATP synthesis and increases the production of reactive oxygen species (ROS) [6]. This, in turn, hinders proper autophagic flux, reducing clearance of damaged organelles and toxic α-synuclein aggregates [7]. Excessive oxidative stress also generates α-synuclein oligomers, which reduce autophagosome formation and alter redox homeostasis, in a vicious cycle, favoring dopaminergic neurodegeneration [8].

Several therapeutic strategies are currently used to counteract neurodegenerative events. Levodopa (L-DOPA), the precursor of dopamine, is the most effective drug used in clinical practice, given its ability to readily cross the blood–brain barrier (BBB) and support dopamine depletion. However, its long-term use leads to L-DOPA-induced dyskinesia (LID) [9]. Hence, such unwanted effects, together with tolerance and differences in patients’ response, emphasize the urgent need for novel therapeutic approaches.

Bromo- and Extra-Terminal domain (BET) proteins (BRD2, BRD3, BRD4, and BRDT) are epigenetic readers capable of interacting with acetylated lysins (Kac) of histone tails and recruiting proteins involved in transcriptional events to promote gene expression. Due to their influence on transcription through chromatin remodeling, BET proteins have emerged as crucial regulators in the expression of genes involved in redox homeostasis, neuroinflammation and autophagy, suggesting a possible involvement in the onset and progression of neurological and non-neurological disorders [10,11,12,13]. Several lines of evidence suggest that BET proteins inhibition may represent a valuable approach to counteract pathomechanisms in various diseases. BET protein inhibition is commonly achieved using small molecules such as JQ1, a selective inhibitor that competitively binds to the bromodomains of BET proteins, thereby preventing their interaction with acetylated lysine residues on histones and transcriptional regulators [14]. Notably, BET protein inhibition by the small molecule JQ1 counteracts redox imbalance and rescues autophagy defects in a preclinical model of Duchenne muscular dystrophy [15]. Additionally, pharmacological blockage of BET proteins attenuates neurodegenerative events by reducing oxidative stress and enhancing autophagy in spinal cord injury and diabetes [16,17]. Nevertheless, the prospective involvement of BET proteins in PD is still elusive.

Here, we present a proof-of-concept study aimed at elucidating possible pathogenic contribution of BET proteins in a cell culture model of PD. To this objective, we employed a loss of function approach by inhibiting the activity of BET proteins through the administration of JQ1. SH-SY5Y and N1E-115 neuronal cells were exposed to the environmental toxin rotenone to mimic the PD phenotype. This experimental model was chosen as it faithfully reproduces most of the PD pathological manifestations. In particular, rotenone has been shown to induce intracellular α-synuclein aggregates reminiscent of Lewy bodies in rodent models [18]; however, such inclusions are absent in cellular systems. Nevertheless, even in vitro, rotenone partially recapitulates alterations in α-synuclein metabolism, as reflected by increased α-synuclein protein levels [19,20,21,22].

We here show that JQ1 administration restores redox homeostasis and autophagic flux, ameliorating neuronal morphology and preventing cell death.

## 2. Materials and Methods

### 2.1. Cell Cultures

Human neuroblastoma SH-SY5Y cells were kindly provided by Dr. Marco Fiocchetti (Roma Tre University) and were originally purchased from the American Type Culture Collection (ATCC; LGC Standards S.r.l., Milan, Italy). Cells were used between passages 10 and 15. Cell line authentication was performed by the originating laboratory prior to distribution, as previously reported [23]. N1E-115 mouse neuroblastoma cells were obtained from the European Collection of Cell Cultures (ECACC; Cat. No. 88112303, Salisbury, UK) and used at passages 15–20.

Both cell lines were cultured at 5% CO_2_ in DMEM medium at high glucose (D6429, Merck Life Science, Milan, Italy), containing 10% (*v*/*v*) fetal bovine serum (FBS, F7524, Merck Life Science, Milan, Italy) and 1% penicillin/streptomycin solution (P06-07100, PAN Biotech, Aidenbach, Germany). For the experiments, 250,000 SH-SY5Y and 150,000 N1E-115 cells were seeded. After 5 h, SH-SY5Y differentiation was induced in DMEM containing 1% FBS and 10 μM retinoic acid (R2625, Merk Life Science, Milan, Italy) for 72 h. SH-SY5Y differentiation was confirmed by the acquisition of a neuronal morphology, characterized by neurite outgrowth, as well as by increased immunoreactivity for the neuronal markers βIII-tubulin and tyrosine hydroxylase (Figure S1A,B). N1E-115 differentiation was achieved by culturing cells in DMEM enriched with 0.5% FBS for 96 h. Differentiated cells were then pre-treated with the BET inhibitor JQ1 (0.1 μM) or vehicle (DMSO, dilution 1:1000 in cell culture media). After 24 h, cells underwent treatment with rotenone (50 nM, R8875, Merck Life Science, Milan, Italy) in the presence or absence of JQ1 for the following 24 h. To evaluate the autophagic flux, specific experiments were set up with chloroquine (20 μM, C6628, Merk Life Science, Milan, Italy) administration in the culture medium for 4 h. Autophagic flux was calculated by subtracting the intensity of LC3-II band in the samples treated with the chloroquine, from the ones without the inhibitor, similarly to what already reported [24].

### 2.2. Immunofluorescence

SH-SY5Y and N1E-115 cells were seeded on sterilized and poly-L-lysine (P6282, Merck Life Science, Milan, Italy)-coated coverslips. Cells were fixed in 4% paraformaldehyde (D1408, Merck Life Science, Milan, Italy) solution in DPBS (D1408, Merck Life Science, Milan, Italy) pH 7.4 for 10 min at RT. Cells were permeabilized with 0.1% Triton X100 (Merck Life Science, Milan, Italy) in DPBS for 5 min. Non-specific binding was blocked by 3% bovine serum albumin (BSA, A3912, Merck Life Science, Milan, Italy) in DPBS containing 0.1% Triton for 1 h at RT. Cells were then incubated overnight with primary antibodies, as reported in Supplementary Table S1. For NOX-4, p22phox and p47phox immunofluorescence, the protocol was performed as follows: (i) 0.05% saponin (558255, Merck Life Science, Milan, Italy) in DPBS for 10 min at RT; (ii) 3% BSA in DPBS for 45 min at RT; (iii) probed overnight with primary antibodies, at 4 °C (Table S1); (iv) incubated with anti-mouse or anti-rabbit fluorescent conjugate antibodies (Table S1), for 1 h at RT; (v) counterstained with DAPI (D9542, Merck Life Science, Milan, Italy); (vi) mounted with Fluoroshield mounting medium (F6182, Merck Life Science, Milan, Italy). Slides were examined by confocal microscopy (TCS SP8, Leica, Wetzlar, Germany) at 40× magnification, and images were acquired with LAS X software (version 3.5.5) (Leica Camera, Wetzlar, Germany) for Windows 10. All acquisition parameters, including laser intensity, photomultiplier gain, and image magnification, were kept unchanged among experimental groups for each immunofluorescence experiment. Protein level quantification was calculated as the mean fluorescence intensity per cell area using ImageJ software v.154d (National Institutes of Health, Bethesda, MD, USA) for Windows 11.

### 2.3. Quantitative Evaluation of Cell Morphology

Morphological evaluation was mainly assessed on brightfield images. Specifically, experiments were conducted as previously reported, and SH-SY5Y cells underwent 72 h rotenone treatment. Images were captured via an inverted confocal microscope (Eclipse 7s100, Nikon Corporation, Tokyo, Japan), and quantitative analysis was performed by ImageJ software v.154d (National Institutes of Health, Bethesda, MD, USA) for Windows 11. Parameters included neurite length (expressed in arbitrary units), neurite-bearing cells (expressed as a percentage calculated from the ratio of the number of neurite-bearing cells to the total cell number per field), neurite network (calculated as the sum of neurite lengths in the field). Morphological assessments were carried out in different experiments, each one involving the analysis of at least three images.

### 2.4. TUNEL Assay

SH-SY5Y were seeded on poly-L-Lysine coated-coverslips. After 24 h JQ1 treatment, oxidative stress was induced via rotenone administration for 48 h. To detect and quantify apoptotic cells, fixed cells were subjected to TUNEL assay (11684795910, Roche, Basilea, Switzerland), following the manufacturer’s instructions.

### 2.5. Evaluation of Enzymatic Activity

To evaluate the activity of pro-oxidant and antioxidant enzymes, NADPH oxidase, superoxide dismutase 1 (SOD1), superoxide dismutase 2 (SOD2), catalase, glutathione reductase (GSR) proteins were considered in this study. Catalase activity was estimated by using a commercial kit (ab83464, Abcam, Cambridge, UK) according to the manufacturer’s instructions. SOD1 activity was measured by following the decrease in the reduction rate of cytochrome c by superoxide radicals in a medium containing 50 mM KH_2_PO_4_ pH 7.8, 20 mM cytochrome c, 0.1 mM xanthine, and 0.01 units of xanthine oxidase, as previously reported [25]. Mitochondrial SOD (SOD2) was assayed following the same procedure in the presence of KCN. GSR activity was measured as previously described [26]. Specifically, the decrease in NADPH absorbance at 340 nm was measured at 30 °C in samples. The reaction mixture contained 0.2 M potassium phosphate buffer, 2 mM EDTA, 2 mM NADPH (in 10 mM Tris-HCl, pH 7), and 20 mM oxidized glutathione (GSSG). The activity was calculated using the NADPH molar extinction coefficient, 6.22 × 10^3^ M^−1^ cm^−1^, considering that one unit of glutathione reductase is defined the amount of enzyme that catalyzes the reduction of 1 μmol of NADPH per minute.

NADPH oxidase activity was assayed according to a modification of the method of Bettaieb et al. [27]. Briefly, cells were homogenized in ice-cold Krebs buffer and then centrifuged at 800× *g*, at 4 °C for 10 min. The supernatant was collected and then centrifuged at 30,000× *g* for 2 h at 4 °C. The pellet (membrane fraction) was resuspended in Krebs buffer, and protein concentration was measured. Aliquots containing 100 μg of protein were added to Krebs buffer containing NADPH (500 μM). The change in absorbance at 340 nm was followed for 10 min at 30 s intervals.

### 2.6. Lysate Preparation and Western Blot Analysis

SH-SY5Y and N1E-115 cells were sonicated in sample buffer (Hepes 10 mM, KCl 10 mM, MgCl_2_ 1.5 mM, NP-40 0.1%, DTT 0.5 mM, protease and phosphatase inhibitor cocktail) for 30 s to obtain a total lysate. Protein concentration was assessed by Lowry’s method and Laemmli buffer was added to denaturate cellular proteins. Then, samples were boiled at 95 °C for 3 min and 20 µg protein extracts were separated on SDS-PAGE electrophoresis. Trans-Blot Turbo system (Biorad Laboratories, Milan, Italy) was used for protein transfer on nitrocellulose membranes, which were then incubated with 5% fat-free milk powder in DPBS containing 0.1% Tween-20 (STS0200, Merck Life Science, Milan, Italy) for 1 h at RT and probed overnight at 4 °C with primary antibodies, as reported in Table S1. Subsequently, membranes were incubated with HRP-conjugated secondary antibodies (Table S1) at RT for 1 h. Clarity ECL Western blotting (1705061, Bio-Rad Laboratories, Milan, Italy) was used to visualize protein-antibody immunocomplexes, and the ChemiDoc MP system (Bio-Rad Laboratories, Milan, Italy) captured the resulting chemiluminescence images. Densitometric analysis was performed using ImageJ software v.154d for Windows 11 (National Institutes of Health, Bethesda, MD, USA). All samples were normalized for protein loading with Vinculin or GAPDH, selected as housekeeping proteins. Densitometric analyses were expressed in arbitrary units, determined by the ratio between the protein band intensity and the respective housekeeping protein.

### 2.7. Dopamine Quantification

Dopamine levels in cell lysates and culture medium were quantified using a Dopamine Competitive ELISA Kit (Thermo Fisher Scientific, Waltham, MA, USA; # EEL144), according to the manufacturer’s instructions.

### 2.8. FIB/SEM Ultrastructural Analysis

SH-SY5Y cells were cultured on coverslips, and fixed with a mixture of 2% formaldehyde and 1% glutaraldehyde in 0.1 M cacodylate buffer for 60 min at 4 °C. Samples were then kept on ice for the following steps, washed in 0.1 M cacodylate buffer (three times for a total of 30 min), and post-fixed in 1% osmium tetroxide in the same buffer for 60 min in the dark. After further washing in buffer, cells were briefly rinsed in distilled water and stained with UranyLess (22409, Electron Microscopy Sciences, Hatfield, PA, USA) for 1 h in the dark. Samples were washed in distilled water (three times for 5 min each), and gradual dehydration was performed in ethanol with 30-min steps at concentrations of 30%, 50%, 75%, 85%, 90%, and 95%. Two 45-min steps in 100% ethanol followed at room temperature. Cells were infiltrated with increasing concentrations of epoxy resin (45359-1EA-F, Sigma-Aldrich, Burlington, MA, USA) in ethanol: 30% resin (two 45-min steps), 70% resin (two 45-min steps), and finally pure resin (two 90-min steps). Excess resin was removed using filter paper, and samples were allowed to polymerize at 60 °C for 72 h. Removal of surplus resin allowed enhanced cell boundary visibility during FIB/SEM observation, facilitating the ion milling process. Coverslips with resin-embedded cells were mounted on stubs using adhesive carbon disks and made conductive by applying a thin gold layer with a K550 sputter coater (Emithech, Kent, UK). Samples were analyzed using a Dual Beam Helios Nanolab 600 system (FEI Company, Hillsboro, OR, USA) at the interdepartmental electron microscopy laboratory (LIME) of Roma Tre University. The samples were sectioned using the ion beam operated at 30 kV and 6.5 nA. Micrographs of the cross-sectioned regions were obtained by detecting backscattered electrons and with the SEM column set at a voltage of 2 kV and a current of 0.17 nA.

### 2.9. Statistical Analysis

Results presented in this study were expressed as mean ± SD (standard deviation). Normal distribution of the data was evaluated by the Shapiro–Wilk test. Unpaired Student’s T-test was performed to compare means between two experimental groups. For three groups comparison, one-way analysis of variance (ANOVA) or two-way ANOVA were carried out, followed by Tukey’s or Bonferroni’s post hoc, respectively. *p* < 0.05 was considered to indicate a statistically significant difference. Statistical analysis and graph editing were carried out using GraphPad Prism 8.4.2 (GraphPad, La Jolla, CA, USA) for Windows 11.

## 3. Results

### 3.1. Expression of BET Proteins in Rotenone-Induced Cell Model of PD

**Figure 1:**
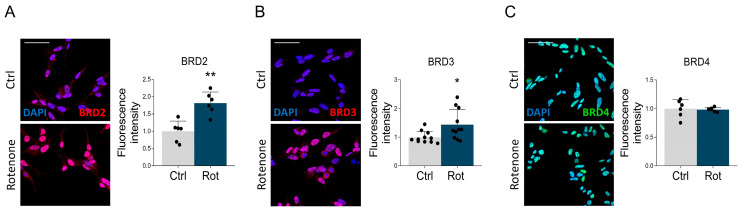
The effect of rotenone administration on BET protein expression. Confocal microscopy representative images and quantitative analysis of (**A**) BRD2 (red), (**B**) BRD3 (red), and (**C**) BRD4 (green) in differentiated SH-SY5Y cells treated with DMSO (Ctrl) and rotenone (Rot, 50 nM) for 24 h. DAPI (blue) was used to counterstain nuclei. Scale bar: 50 µm. Data are represented as means ± SD. The black dots depicted around the SD represent individual values, each derived from the average fluorescence calculated in the cells present in a single image (each image derives from a different experiment). *N* = 6–11 biological replicates. Statistical analysis was performed by using the Unpaired Student’s *t*-test. * *p* < 0.05; ** *p* < 0.01.

Based on the knowledge that the expression of BET proteins may change in neurological conditions [10], we evaluated possible alterations in the protein expression of BRD2, BRD3, and BRD4 in the parkinsonian phenotype induced by 24-h rotenone administration to differentiated SH-SY5Y cells. Immunofluorescence analysis revealed localization of BET proteins mostly restricted to the nucleus, marked with DAPI. Interestingly, rotenone stimulation significantly increased BRD2 and BRD3 immunoreactivity (Figure 1A,B), while no changes were observed for BRD4 (Figure 1C).

### 3.2. BET Blockade Attenuates the Parkinsonian Phenotype and Counteracts Oxidative Stress

The altered expression of BRD2 and BRD3 following rotenone treatment further supports the hypothesis that deregulation of BET proteins may concur to PD physiopathology, leading us to investigate the effects induced by the selective BET inhibitor JQ1.

**Figure 2:**
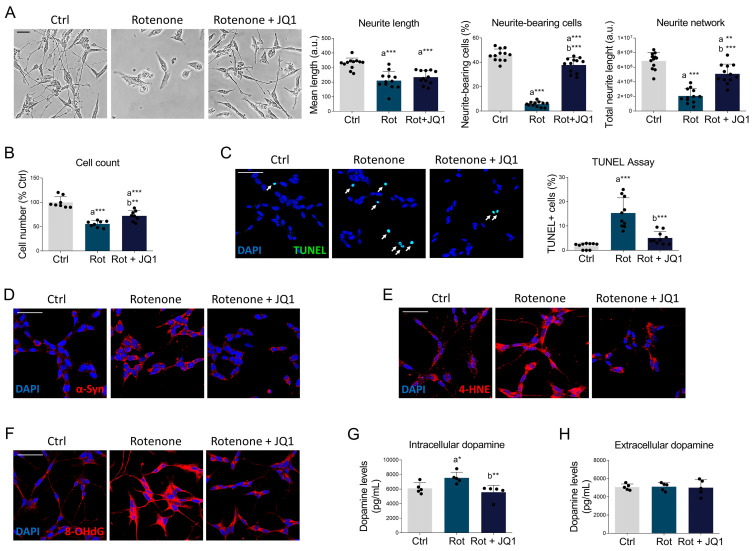
JQ1 treatment ameliorates the pathological hallmarks of Parkinson’s disease. (**A**) Representative brightfield images and respective quantitative analysis of neuronal morphology parameters (neurite length, percentage of neurite bearing cells, and neurite network) of differentiated SH-SY5Y treated with DMSO (Ctrl), rotenone (Rot, 50 nM) or rotenone with JQ1 (Rot + JQ1, 100 nM) for 24 h. *N* = 12 biological replicates. (**B**) Cell count of SH-SY5Y cells treated as in (**A**). *N* = 8 independent experiments. (**C**) TUNEL assay (green) of differentiated SH-SY5Y cells treated as previously described. Nuclei were counterstained with DAPI (blue). *N* = 10 biological replicates. White arrows indicate TUNEL-positive nuclei. (**D**–**F**) Immunofluorescence of α-syn (red), 4-HNE (red), and 8-OH(d)G (red) in differentiated SH-SY5Y treated as reported above. DAPI (blue) was used to counterstain nuclei. *N* = 5–8 independent experiments. Quantitative analysis is reported in Supplementary Figure S2. Data are represented as means ± SD. (**G**) Intracellular and (**H**) extracellular dopamine levels estimated by ELISA assay in SH-SY5Y treated as above. Dopamine concentrations are expressed as pg/mL in cell lysates or culture media. *N* = 5 biological replicates. The black dots around the SD represent the different biological measurements. Statistical analysis was performed by using one-way ANOVA followed by Tukey’s post hoc test. “a” indicates statistical significance vs. Ctrl; “b” indicates statistical significance vs. rotenone group. * *p* < 0.05, ** *p* < 0.01, *** *p* < 0.001. Scale bar: 50 µm.

Rotenone treatment caused neurite retraction in differentiated SH-SY5Y cells, as assessed by measuring single neurite length and the percentage of neurite-bearing cells. Consequently, a dramatic reduction in the neurite network (i.e., total neurites length in a given field) was observed. Interestingly, JQ1 mitigated all these effects of rotenone (Figure 2A). The parkinsonian phenotype was also accompanied by a significant reduction in cell number, partially prevented by JQ1 administration (Figure 2B). Such neuronal loss was indeed due to apoptotic induction, as evidenced by the increased number of TUNEL-positive cells, which was counteracted by BET inhibition with JQ1 (Figure 2C).

In addition, JQ1 treatment abolished the rotenone-induced abnormal accumulation of α-synuclein, a neuropathological hallmark of PD (Figure 2D and Figure S2A). Since oxidative stress, associated with mitochondrial dysfunction, is considered a hallmark of PD neuropathology [5,28], we explored immunoreactivity levels of 4-hydroxynonenal (4-HNE), a marker of lipid peroxidation (Figure 2E and Figure S2B), and of 8-hydroxy-(deoxy)guanosine (8-OH(d)G), a marker of oxidative damage to nucleic acids (Figure 2F and Figure S2C). The latter was mostly confined to the cytoplasm, according to the notion that oxidative damage in PD neurons primarily affects RNA and mitochondrial DNA [29]. Notably, BET blockade by JQ1 significantly blunted oxidative stress (Figure 2E,F and Figure S2B,C). Deregulation of dopamine metabolism is another hallmark of PD. Consistent with previous studies [30], rotenone exposure increased intracellular dopamine levels (Figure 2G), without affecting the amount of dopamine released into the culture medium (Figure 2H). Notably, BET inhibition restored intracellular dopamine content to levels comparable to those observed in control cells (Figure 2G).

**Figure 3:**
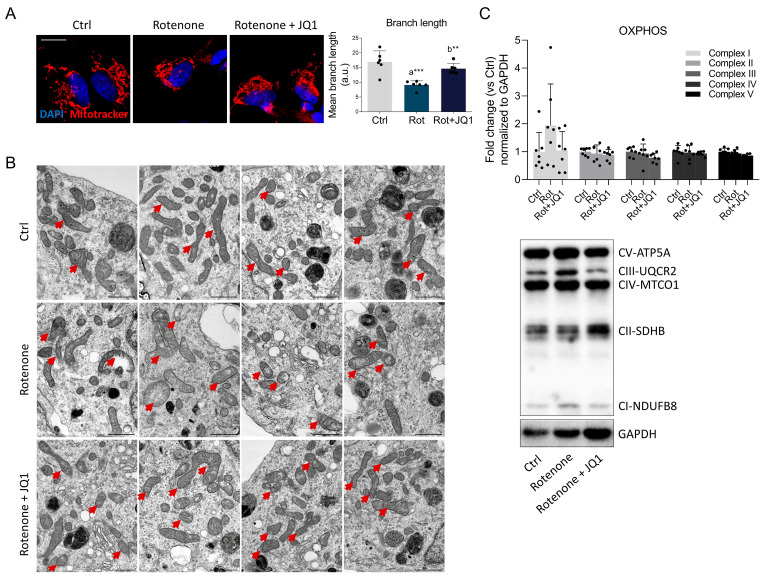
BET blockage by JQ1 improves rotenone-induced mitochondrial abnormalities. (**A**) Mitochondria visualization and quantification of mitochondrial branch length were assessed by MitoTracker staining of differentiated SH-SY5Y treated with DMSO (Ctrl), rotenone (Rot, 50 nM) or rotenone with JQ1 (Rot + JQ1, 100 nM) for 24 h. DAPI (blue) was employed to counterstain cell nuclei. *N* = 6 independent experiments. Scale bar: 10 µm. (**B**) Representative FIB/SEM micrographs depicting the mitochondrial morphology of differentiated SH-SY5Y cells subjected to the three experimental conditions examined. Scale bars, 1 µm. Red arrowheads indicate representative mitochondria. (**C**) Representative Western blot and densitometric analysis of CI-NDUFB8, CII-SDHB, CIII-UQCRC2, CIV-MTCO1, and CV-ATP5A in SH-SY5Y treated as aforementioned. GAPDH was used for loading control. Data are represented as means ± SD. The black dots around the SD represent the different biological measurements. *N* = 7 different biological replicates. Statistical analysis was performed using one-way ANOVA followed by Tukey’s post hoc test. “a” indicates statistical significance vs. Ctrl; “b” indicates statistical significance vs. rotenone group. ** *p* < 0.01, *** *p* < 0.001.

Considering that mitochondrial homeostasis is altered in PD [31], we used MitoTracker to visualize mitochondrial mass and networks. Despite the absence of any significant variation in mitochondrial mass (Figure S3A), mitochondrial networks were found to be markedly fragmented in rotenone-treated SHSY5Y cells, with the appearance of donut/blob-shaped structures, as well as a reduction in the mean branch length. In contrast, JQ1 abrogated such abnormalities (Figure 3A).

These results prompted us to investigate mitochondrial ultrastructure using focused ion beam scanning electron microscopy (FIB/SEM). We explored possible variations in the mitochondrial number across the three conditions; however, no significant differences were found (Figure S3B). Nevertheless, qualitative variations in mitochondrial features were detected (Figure 3B). In control cells, mitochondria exhibited a slender and regular shape, with a dense matrix and regularly arranged cristae. In contrast, rotenone-treated cells showed mitochondrial matrix rarefaction and disorganization of inner membranes, with deranged and fragmented cristae. In the most severe cases, mitochondria showed ruptures in their outer membrane and swelling. Interestingly, treatment with JQ1 partially restored mitochondrial morphology attenuating cristae loss, preventing fragmentation, and mitigating swelling and matrix rarefaction, highlighting a potential role for BET inhibition in preserving mitochondrial integrity. Western blot analysis revealed that the expression of proteins belonging to oxidative phosphorylation (OXPHOS) did not vary significantly between the experimental groups (Figure 3C). However, complex I (NDUFB8) exhibited a non-significant tendency to increase in the rotenone-treated group, which could be indicative of an attempt to compensate for the inhibition of complex I activity promoted by the neurotoxin. Nevertheless, this trend was prevented by BET pharmacological inhibition.

### 3.3. BET Inhibition Regulates the Expression of the Master Regulators Controlling Oxidative Stress and Mitochondrial Homeostasis

**Figure 4:**
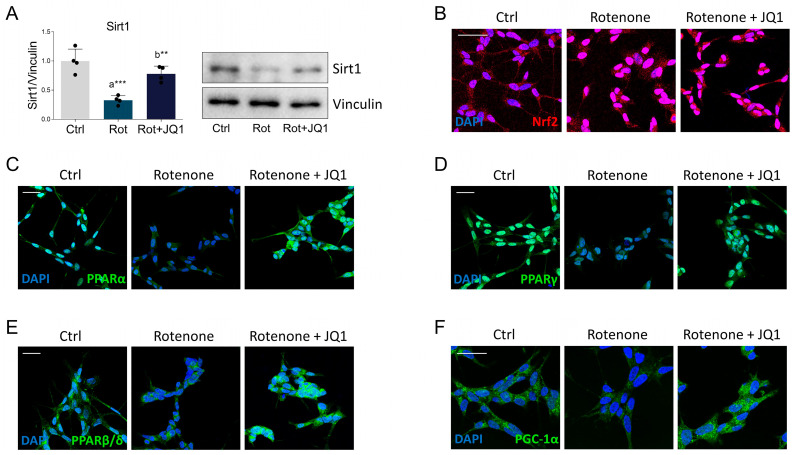
Impact of JQ1 on the expression of transcriptional regulators controlling redox and mitochondrial homeostasis. (**A**) Differentiated SH-SY5Y cells were treated with DMSO (Ctrl), rotenone (Rot, 50 nM) or rotenone with JQ1 (Rot + JQ1, 100 nM) for 24 h and immunoblot assay was performed to evaluate the expression of SIRT1. Vinculin was used for loading control. Data are represented as means ± SD. The black dots around the SD represent the different biological measurements. *N* = 4 biological replicates. Immunofluorescence of (**B**) Nrf2 (red), (**C**) PPARα (green), (**D**) PPARγ (green), (**E**) PPARβ/δ (green) and (**F**) PGC-1α (green) in SH-SY5Y differentiated cell line treated as previously described. DAPI (blue) was used for nuclei staining; immunofluorescence signal quantifications are reported in Supplementary Figure S4. Statistical analysis was performed using one-way ANOVA followed by Tukey’s post hoc test. “a” indicates statistical significance vs. Ctrl; “b” indicates statistical significance vs. rotenone group. ** *p* < 0.01, *** *p* < 0.001. Scale bar: 50 µm.

Based on the previous results, we evaluated the expression of the key regulators of mitochondrial homeostasis and redox metabolism. Silent information regulator sirtuin 1 (Sirt1) is a deacetylase whose activity is closely linked to the regulation of mitochondrial function and oxidative stress. Specifically, Sirt1 activity and expression are suppressed in PD, increasing the vulnerability of neurons to cell damage [32]. Immunoblot analysis shows that JQ1 properly prevented the dramatic decrease in Sirt1 expression following rotenone treatment (Figure 4A). We next examined the expression and localization of nuclear factor erythroid 2-related factor 2 (Nrf2), a transcriptional regulator crucially involved in the regulation of mitochondrial quality control and redox metabolism [33]. As expected, ROS overproduction induced by rotenone treatment led to Nrf2 stabilization and accumulation, particularly at the nuclear level, which was unaffected by BET inhibition (Figure 4B and Figure S4A).

Attention then turned to the expression of the peroxisome proliferator-activated receptors (PPAR). These transcription factors orchestrate key neuroprotective pathways, countering mitochondrial dysfunction, oxidative stress, and neuroinflammation [34]. BET inhibition by JQ1 effectively attenuates the rotenone-mediated reduction in PPARα and PPARγ (Figure 4C,D and Figure S4B,C). As previously reported [35], both transcription factors are primarily located in the nucleus. However, there was a notable increase in PPARα immunoreactivity also at the cytosolic level, suggesting that JQ1 may promote the accumulation of the newly synthesized protein. According to other findings [36], PPARβ/δ is present in both the nucleus and cytosol of neuronal cells. Notably, its levels were not affected by rotenone treatment. However, JQ1 significantly increased PPARβ/δ immunoreactivity, similar to what was observed for the other PPAR isoforms (Figure 4E and Figure S4D).

The activity of Nrf2 and PPARs may depend on the presence of the peroxisome proliferator-activated receptor gamma coactivator 1-alpha (PGC-1α) [33]. Our studies showed that PGC-1α expression was significantly reduced following rotenone stimulation (Figure 4F and Figure S4E), supporting evidence that this transcriptional coactivator is significantly downregulated in PD tissues and negatively correlated with the disease severity [37]. Conversely, BET blockade by JQ1 completely abolished the altered PGC-1α expression elicited by the parkinsonian phenotype (Figure 4F and Figure S4E). Although PGC-1α localization is generally nuclear, immunofluorescence revealed the appearance of an intense dot-like staining, mainly confined to the cytoplasm. This peculiar feature has already been observed in neuronal cells and may reflect the presence of PGC-1α inside the mitochondria, where it interacts with the mitochondrial transcription factor A (TFAM) to increase the expression of mitochondrial genes [38].

### 3.4. BET Inhibition by JQ1 Promotes the Antioxidant Response and Suppresses the Pro-Oxidant NADPH Oxidase Complex in Rotenone-Treated SH-SY5Y Cells

**Figure 5:**
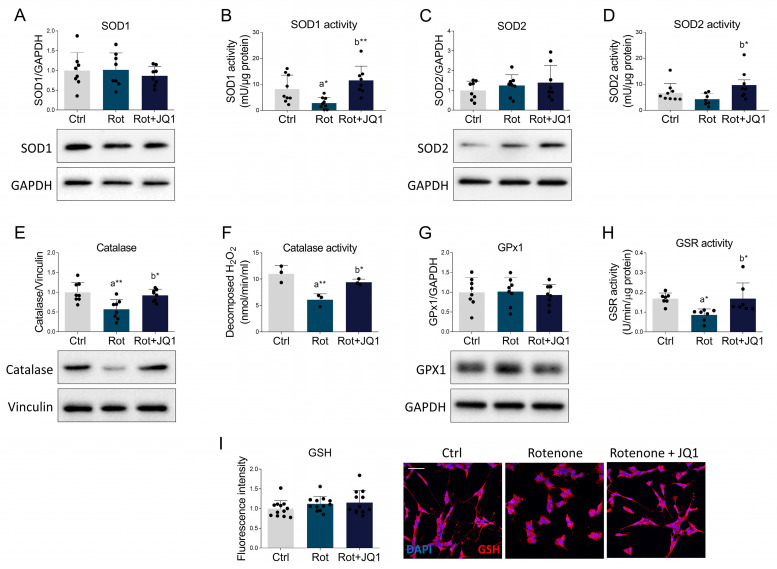
BET blockade ameliorates the antioxidant response in the parkinsonian phenotype. Differentiated SH-SY5Y cells were treated with DMSO (Ctrl), rotenone (Rot, 50 nM) or rotenone with JQ1 (Rot + JQ1, 100 nM) for 24 h. (**A**) Representative Western blot and densitometric analysis of SOD1. GAPDH was used as housekeeping protein for loading control. *N* = 8 independent experiments. (**B**) SOD1 activity, expressed as milliunit per µg of protein lysate. *N* = 9 experimental replicates. (**C**) Representative Western blot and densitometric analysis of SOD2. GAPDH was used as loading control to normalize protein levels. *N* = 8 biological replicates. (**D**) SOD2 activity, expressed as milliunit per µg of protein lysate. *N* = 9 different experiments. (**E**) Representative Western blot and densitometric analysis of catalase. Vinculin was chosen as loading control. *N* = 8 biological replicates. (**F**) Catalase activity, expressed as the amount of decomposed hydrogen peroxide (nanomoles produced in 1 min from 1 mL of cell lysate). *N* = 3 independent experiments. (**G**) Representative Western blot and densitometric analysis of GPx1. *N* = 8 biological replicates. GAPDH was used as housekeeping protein. (**H**) GSR activity, expressed as units in 1 min per 1 mg of total cell lysate. *N* = 7 different replicates. (**I**) Confocal microscopy showing immunoreactivity of GSH (red). Nuclei were counterstained with DAPI (blue). *N* = 12 independent experiments. Scale bar: 50 µm. Data are represented as means ± SD. The black dots around the SD represent the different biological measurements. Statistical analysis was performed by using one-way ANOVA followed by Tukey’s post hoc test. “a” indicates statistical significance vs. Ctrl; “b” indicates statistical significance vs. rotenone group. * *p* < 0.05, ** *p* < 0.01.

To elucidate the molecular mechanisms underlying the beneficial effects of BET inhibition on oxidative stress, we investigated the impact of JQ1 on the main enzymes involved in the cell antioxidant defense. Superoxide dismutases (SODs) are responsible for catalyzing the dismutation of superoxide anion to hydrogen peroxide and molecular oxygen [39]. Although SOD1 protein expression was unchanged (Figure 5A), its enzymatic activity was significantly reduced by rotenone (Figure 5B), reinforcing evidence that parkinsonism is characterized by the presence of dysfunctional SOD1 [40].

Importantly, BET blockade properly restored SOD1 function (Figure 5B). Likewise, although SOD2 protein expression was unaltered in the experimental groups considered in this study (Figure 5C), the enzymatic activity was enhanced by JQ1 treatment (Figure 5D). Catalase degrades hydrogen peroxide to oxygen and water in a two-step reaction [41]. Immunoblot analysis revealed that BET blockade by JQ1 prevented the rotenone-induced reduction in catalase expression (Figure 5E). Consistently, JQ1 also abolished the dysfunctional enzymatic activity evoked by the neurotoxin (Figure 5F). In contrast, no differences were detected in the expression of glutathione peroxidase 1 (GPx1) (Figure 5G), which reduces hydrogen peroxide to water via GSH [42]. Glutathione reductase (GSR) catalyzes the reduction in glutathione disulfide, thereby regenerating the pool of reduced glutathione (GSH) required to maintain the antioxidant defense of the cell [43]. Our results indicate that rotenone significantly suppressed GSR activity (Figure 5H), which is in line with what was already observed in PD neurons [44]. On the other hand, pharmacological BET inhibition completely reversed GSR dysfunction (Figure 5H). S-glutathionylation is a reversible event that protects the target proteins from oxidative damage [45]. However, although changes in the expression of glutathione S-transferase enzymes have been reported in PD models [46], protein S-glutathionylation was not affected by either rotenone or JQ1 in our experimental setup (Figure 5I).

**Figure 6:**
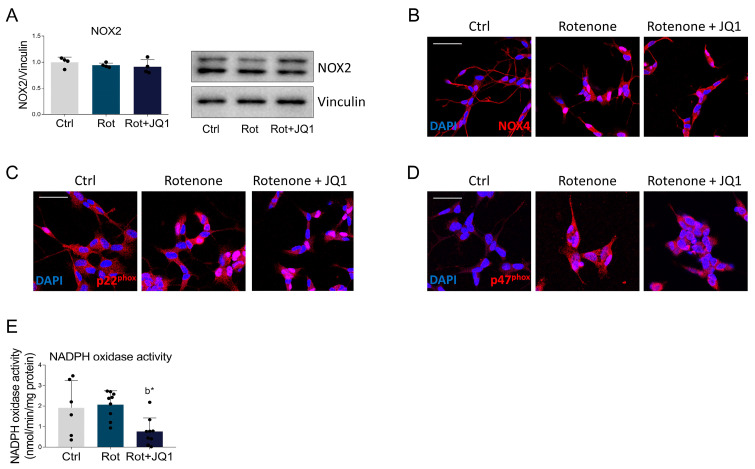
Effects of BET inhibition on NADPH oxidase complex in rotenone-treated SH-SY5Y cells. (**A**) Representative Western blot and densitometric analysis of NOX2 subunit in cells treated with DMSO (Ctrl), rotenone (Rot, 50 nM) or rotenone with JQ1 (Rot + JQ1, 100 nM) for 24 h. Vinculin was chosen as loading control. *N* = 4 biological replicates. Representative immunofluorescence images of (**B**) NOX4 (red), (**C**) p22phox (red) and (**D**) p47phox (red) NADPH oxidase subunits. DAPI (blue) was used to counterstain cell nuclei. *N* = 6–12 experimental replicates. Quantitative analysis is reported in Supplementary Figure S5. Scale bar: 50 µm. (**E**) Enzymatic activity assay of NADPH oxidase complex performed on SH-SY5Y cells treated as described above. *N* = 6–9 independent experiments. Data are expressed as mean ± SD. The black dots around the SD represent the different biological measurements. Statistical analysis was performed using the one-way ANOVA test, followed by Tukey’s post hoc test. “b” indicates statistical significance vs. rotenone experimental group. Statistical significance is indicated as follows: * *p* < 0.05.

Intracellular redox homeostasis is maintained by a continuous balance between antioxidant enzymes and pro-oxidant systems [47]. Among the ROS-generating enzymes, NADPH oxidase has been implicated in PD pathogenesis [48]. While no alterations in the expression of the NADPH oxidase subunits NOX2, NOX4 and p22phox were observed (Figure 6A–C and Figure S5A,B), the immunoreactivity of the p47phox subunit was higher in the rotenone-treated group and remained unchanged upon JQ1 administration (Figure 6D and Figure S5C). To further investigate the putative involvement of NADPH oxidase, we also performed an enzymatic assay and showed that while rotenone did not affect the activity of the pro-oxidant complex, BET blockade significantly reduced its activity (Figure 6E).

Acyl-CoA oxidase 1 (ACOX1) is the rate-limiting enzyme in peroxisomal β-oxidation, producing H_2_O_2_ as by-product [49]. To assess whether the increased oxidative stress observed in our cell culture model of PD might also depend on prospective changes in this enzyme, we performed Western blot analysis. The data collected demonstrated that ACOX1 protein expression was not altered by either rotenone or JQ1 (Figure S5D).

Taken together, these results highlight that BET blockade suppresses rotenone-induced oxidative stress by increasing the activity of antioxidant enzymes and suppressing the activation of the pro-oxidant NADPH oxidase complex.

### 3.5. BET Blockade by JQ1 Attenuates Autophagy Impairment in the Rotenone-Induced Cell Model of PD

**Figure 7:**
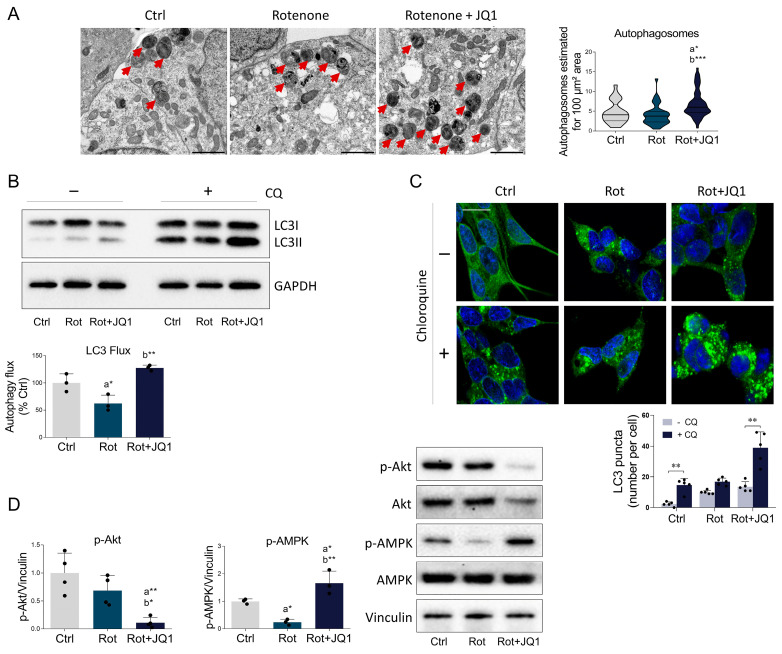
BET inhibition by JQ1 ameliorates autophagy alterations induced by rotenone. (**A**) FIB/SEM micrographs illustrating autophagosomes in differentiated SH-SY5Y cells treated with DMSO (Ctrl), rotenone (Rot, 50 nM), and rotenone with JQ1 (Rot + JQ1, 0.1 μM) for 24 h. Scale bar: 2 µm. Red arrowheads indicate representative autophagosomes. (**B**) Immunoblot analysis and densitometric analysis of LC3 showing autophagy flux. Differentiated SH-SY5Y were treated as in (**A**) and chloroquine (20 µM) was added 4 h before the end of the experiment. GAPDH was used as internal loading control. *N* = 3 independent experiments. (**C**) Immunofluorescence of LC3 (green) and mean number of autophagosomes per cell in SH-SY5Y treated as in (**B**). Nuclei were counterstained with DAPI (blue). *N* = 5 independent experiments. Scale bar: 10 µm. (**D**) Representative immunoblot and densitometric analysis of phosphorylated Akt (Ser473) and phosphorylated AMPK (Thr172) in the three experimental conditions examined. Vinculin served as loading control. *N* = 3–4 independent experiments. Data are represented as mean ± SD. The black dots around the SD represent the different biological measurements. Statistical analysis was carried out by using one-way ANOVA, followed by Tukey’s post hoc or two-way ANOVA, followed by Bonferroni’s post hoc. “a” indicates statistical significance vs. Ctrl; “b” indicates statistical significance vs. rotenone group. * *p* < 0.05; ** *p* < 0.01; *** *p* < 0.001.

Several lines of evidence suggest that autophagy dysfunction may significantly contribute to the pathological phenotype of PD. In line with this notion, autophagy dysregulation has been described in various neurotoxic experimental models of PD, including the rotenone-mediated parkinsonian phenotype [50]. Therefore, we investigated the prospective effects of BET inhibition on autophagy in rotenone-treated SH-SY5Y cells. Immunoblot analysis revealed that the protein level of Beclin1, a key player in autophagosome formation and maturation, was unaffected (Figure S6A). We then analyzed the expression of LC3, a selected marker of autophagic vacuoles. LC3II, corresponding to the active lipidated form, was significantly upregulated after JQ1 administration (Figure S6B). The ultrastructural analysis corroborated these findings, revealing a substantial increase in the number of autophagic compartments in JQ1-treated cells compared to control and rotenone-treated groups (Figure 7A).

Nevertheless, the morphological analysis and data related to LC3 appeared of complex interpretation, as both the upregulation of LC3II and the increase in the number of autophagic vacuoles could indicate either an enhancement in autophagic activity or an impairment of lysosomal degradation leading to the accumulation of late-stage autophagosomes [50]. To gain a clearer insight into the biological significance of the observed changes in lipidated LC3II accumulation and the rise in autophagic vacuoles, we performed an autophagy flux experiment by using chloroquine (CQ), a lysosomotropic agent capable of inhibiting the autophagy flux by blocking the fusion of autophagosomes with lysosomes [51]. Using LC3II basal levels as a reference, CQ administration induced a lower buildup in LC3II levels in rotenone-treated cells compared to control or JQ1-treated cells. Accordingly, densitometric analysis showed that BET inhibition completely prevented the reduction in the autophagy flux induced by the neurotoxin (Figure 7B). These results were further supported by immunofluorescence analysis. CQ treatment led to an obvious appearance of autophagosomes in control cells, while it failed to significantly increase the number of LC3 dots in rotenone-treated cells, indicating a slower autophagic flux. In contrast, a significant increase in the number of LC3 puncta was observed upon administration of CQ to JQ1-treated cells (Figure 7C), suggesting that pharmacological inhibition of BET proteins counteracts the autophagy blockade seen in the PD phenotype.

To elucidate the molecular mechanisms explaining the changes in the autophagy process, we then analyzed the two main kinases that regulate autophagy activation. Akt activation abolishes autophagy by inhibiting proteins involved in autophagy induction [52]. Conversely, AMPK activation triggers autophagy by promoting the activating phosphorylation of several proteins belonging to the autophagic machinery [53]. Although no differences were detected between control and rotenone-treated cells, JQ1 administration significantly dampened the activating phosphorylation of Akt. Additionally, rotenone exposure resulted in a significant reduction in the activation state of AMPK, an effect that was completely prevented by BET blockade (Figure 7D).

Overall, these data underline that autophagy flux is impaired upon rotenone exposure, as observed in other PD models, and that this effect is mainly due to the suppression of AMPK activity [54]. Importantly, BET inhibition properly restores autophagy by suppressing the Akt axis and enhancing the activation state of AMPK.

### 3.6. JQ1-Mediated Neuroprotective Effects Are Recapitulated in Another Cell Model of PD

**Figure 8:**
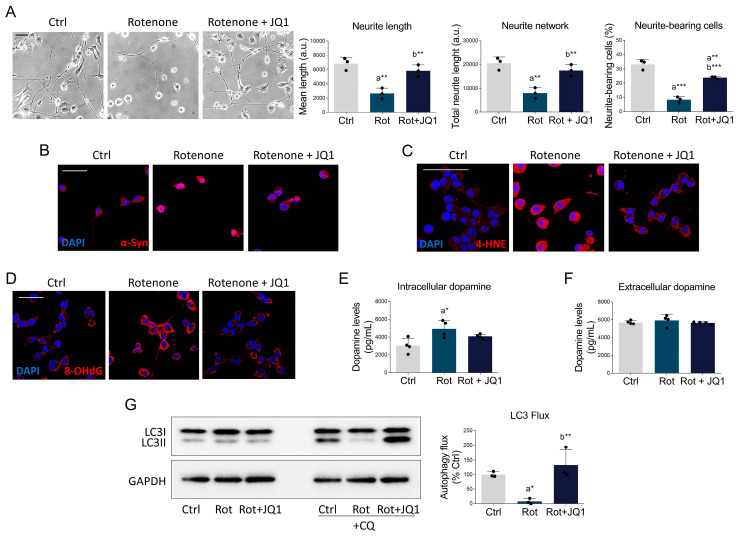
BET inhibition protects against the parkinsonian phenotype in N1E-115 cells. (**A**) Representative brightfield images and quantitative assessment of neuronal morphology (neurite length, neurite network, and percentage of neurite-bearing cells) of differentiated N1E-115 neuronal cells treated with DMSO (Ctrl), rotenone (Rot, 50 nM), and rotenone with JQ1 (Rot + JQ1, 0.1 μM) for 24 h. *N* = 3 biological replicates. Scale bar: 50 µm. Representative immunofluorescence images of (**B**) α-synuclein (red), (**C**) 4-HNE (red), and (**D**) 8-OHdG (red) in differentiated N1E-115 neuronal cells treated as previously described. DAPI (blue) was used to counterstain nuclei. Scale bar: 50 µm. *N* = 3 different biological replicates. (**E**) Intracellular and (**F**) extracellular dopamine were estimated by ELISA assay in N1E-115 treated as above. Dopamine levels are expressed as pg/mL in cell lysates or culture media. *N* = 4 biological replicates. (**G**) Representative Western blot of LC3 (left) and calculation of the autophagy flux (right) in N1E-115 cells treated as in A, with or without chloroquine (20 μM) for 4 h. Data are expressed as mean ± SD. The black dots around the SD represent the different biological measurements. *N* = 3 different biological replicates. Statistical analysis was performed by using one-way ANOVA, followed by Tukey’s post hoc. “a” indicates statistical significance vs. Ctrl; “b” indicates statistical significance vs. rotenone group. * *p* < 0.05; ** *p* < 0.01; *** *p* < 0.001.

To exclude that the effects observed in SH-SY5Y were dependent on the specific background of the selected cell line, we summarized the main findings in another cell culture model by using N1E-115 cells. N1E-115 is a murine neuroblastoma cell line that is capable of acquiring neuronal-like properties upon appropriate stimuli, making it a suitable model for studying dopamine metabolism and PD physiopathology [55,56]. Therefore, N1E-115 cells were differentiated in low serum (0.5% FBS) for three days and treated with rotenone to induce PD neuropathology. Similarly to what previously observed in SH-SY5Y, rotenone administration increased BRD2 (Figure S6C) and BRD3 (Figure S6D) immunoreactivity, whereas no changes were observed for BRD4 (Figure S6E). Notably, BET inhibition by JQ1 significantly prevented the alterations in cell morphology induced by the environmental toxin (Figure 8A).

BET blockade also blunted the increased immunopositivity for α-synuclein observed in rotenone-treated N1E-115 (Figure 8B). Immunostaining for oxidative stress markers was particularly intense after rotenone administration but was significantly attenuated by JQ1 (Figure 8C,D). Abnormally elevated intracellular dopamine content was also reduced by BET inhibition (Figure 8E), whereas released dopamine levels remained unaffected across all experimental conditions (Figure 8F). In addition, pharmacological BET inhibition completely prevented the dysfunction in the autophagy process, which was found to be nearly suppressed after rotenone stimulation (Figure 8G).

## 4. Discussion

The complex PD pathogenesis is multifactorial, involving aging, genetic mutations, and environmental exposure to toxins [57]. In addition, the clinical management of PD is complicated by the numerous alterations in molecular mechanisms contributing to the pathological phenotype, such as aberrant protein aggregation, oxidative stress, mitochondrial dysfunction, and defective autophagy [58]. To date, available therapies for PD offer only limited benefits and do not effectively counteract the dramatic loss of dopaminergic neurons. Therefore, future research must address the need for novel approaches able to efficiently target multiple aspects of the disease.

In this context, pharmacological modulation of BET proteins could represent a novel and promising strategy to improve neuronal resilience in PD. Indeed, a growing body of evidence suggests that BET inhibition reduces oxidative stress and restores autophagy in several pathological conditions [15,16]. Furthermore, BET blockade also suppresses the inflammatory response in several brain disorders [13]. Despite this notion, the role of BET modulation in PD is so far unexplored.

**Figure 9:**
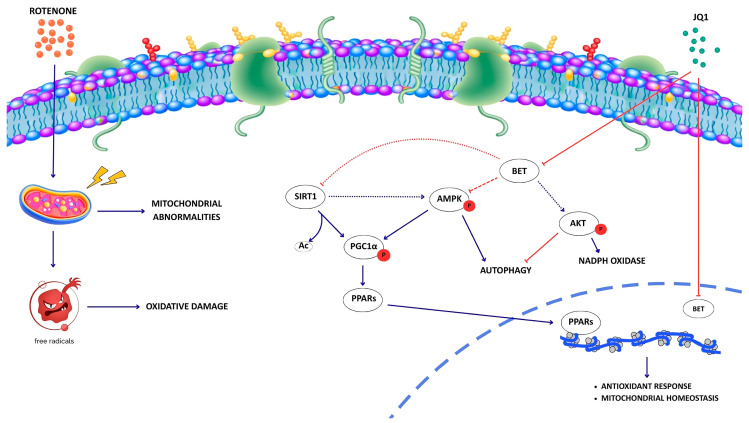
Working model depicting the proposed molecular mechanisms underlying the neuroprotective effect of JQ1 against rotenone-induced cytotoxicity. Blue arrows indicate activation, red arrows indicate inhibition. Dashed arrows indicate indirect regulation or pathways whose underlying mechanisms are not yet fully elucidated.

In this proof-of-concept study, we provide evidence that rotenone administration is accompanied by the increase in BRD2 and BRD3 protein levels in both SH-SY5Y and N1E-115 cells. Although BRD2, BRD3 and BRD4 belong to the same protein family, increasing evidence indicates that they may exert non-redundant and context-dependent functions [59,60]. In line with this notion, the selective increase in BRD2 and BRD3 following rotenone treatment, in the absence of changes in BRD4 expression, may reflect differential transcriptional regulation or protein stabilization mechanisms acting on individual BET members. The lack of changes in BRD4 expression does not exclude its involvement in the transcriptional alterations associated with the pathological condition. Indeed, BRD4 activity may be enhanced through increased recruitment to chromatin, potentially driven by alterations in histone acetylation, a feature that has been reported in PD [61,62]. Consistently, previous studies in other pathological contexts have shown that BET protein recruitment to chromatin can be increased independently of changes in protein abundance [15]. Although Chromatin ImmunoPrecipitation (ChIP) assays would be required to directly address this hypothesis, these considerations support a broader involvement of BET proteins in PD-related transcriptional dysregulation beyond mere expression changes. Beyond the specific involvement of the individual BET proteins, our data indicate that pharmacological inhibition attenuates the parkinsonian phenotype in rotenone-based cell models of PD, possibly through the coordination of multiple cellular processes (Figure 9). Specifically, JQ1 treatment significantly reduces cell death, morphological alterations, and α-synuclein accumulation elicited by rotenone in dopaminergic SH-SY5Y and N1E-115 neuronal cells. Importantly, BET inhibition normalizes the intracellular accumulation of dopamine induced by rotenone. Dopamine accumulation is consistent with previous reports showing that PD models, and possibly human PD brains, are characterized by alterations in dopamine metabolism, resulting in intracellular buildup and impaired extracellular release. Notably, aberrant intracellular dopamine accumulation has been shown to contribute to ROS generation and dopaminergic neuronal cell death, further exacerbating neurodegenerative processes [30,63,64,65,66,67,68].

In addition, BET blockade also protects mitochondrial morphology. These effects are associated with significant restoration of the cellular antioxidant defense machinery. Although these enzymes are able to cope with moderate ROS levels, SOD1, SOD2, catalase and GSR activities are reduced by rotenone administration. Indeed, excessive oxidative stress can either reduce the transcription of antioxidant enzymes or favor the destabilization of their structure, leading to enzymatic dysfunction [69,70]. Whilst the molecular mechanisms still need to be elucidated, BET inhibition efficiently rescued activity of SOD1, SOD2, catalase and GSR. In addition, BET blockade also restores the expression of catalase: such effect can be attributed to the upregulation of Sirt1, PPARα, PPARβ/δ, PPARγ and PGC-1α, which are critical transcriptional regulators of the antioxidant response.

Oxidative stress in PD is also promoted by the NADPH oxidase complex. Several lines of evidence have reported increased expression of NAPDH oxidase subunits in human PD brains and experimental models of the disease [71]. Although the activity of the NADPH oxidase complex was unchanged in rotenone-treated cells, we observed a significant increase in p47phox expression in our PD model, as reported in the literature [72]. We have previously shown that BET proteins directly associate with chromatin regulatory regions of the NADPH oxidase subunits in skeletal muscle, and that JQ1 administration significantly reduces BRD2 and BRD4 at these regions, leading to suppression of gene transcription [15]. Since BET blockade did not alter the expression of any of the NADPH oxidase subunits in rotenone-treated SH-SY5Y, it is reasonable to speculate that the transcriptional control of these subunits by BET proteins differs in various physiopathological contexts, and that the decreased NADPH oxidase activity that we detected may have been induced by indirect post-translational mechanisms.

Increased ROS levels have been associated with dysfunctional autophagy in several physiopathological contexts, including PD [15,48,73]. Specifically, rotenone-induced autophagy failure depends on redox modification of autophagy-related proteins, deregulation of upstream signaling pathways, and decreased lysosomal activity [48,73]. In our PD model, BET inhibition significantly restores autophagy flux. This result is consistent with what was observed in other pathological conditions [15]. Notably, we provide evidence that JQ1-mediated induction of autophagy is associated with modulation of both Akt and AMPK activity. Previous data suggested that BET proteins, particularly BRD4, enrich the promoters of several tyrosine kinase (RTK) receptors and their recruitment leads to the upregulation of RTK expression and subsequent induction of the PI3K-Akt axis. Conversely, BET displacement by JQ1 blocks the activation of this signaling cascade [74]. In addition to the effect on the autophagic process, the JQ1-dependent decrease in Akt may also explain the reduced activity of NADPH oxidase shown in this study, since the activation of this pro-oxidant complex depends on the site-specific phosphorylation of p47phox by Akt [75]. BET inhibition also counteracts ROS-induced downregulation of AMPK activity, likely through a mechanism involving Sirt1 [15]. ROS affects Sirt1 stability, promoting its post-translational modification and its subsequent degradation [76]. Thus, in this study, the susceptibility to oxidative damage is consistent with the reduced Sirt1 expression mediated by rotenone, similar to what has been previously reported in other PD models [77,78]. Importantly, the decrease in Sirt1 expression favors LKB1 acetylation, inhibiting its ability to operate the activating phosphorylation on AMPK [79]. JQ1 treatment significantly increases Sirt1 levels and AMPK activation in rotenone-treated cells, supporting the notion that the Sirt1/AMPK pathway may contribute to the restoration of autophagy induced by BET blockade in the PD phenotype.

Rescue of autophagic flux might explain the reduction in α-synuclein accumulation following BET pharmacological inhibition, given that α-synuclein is degraded both by the proteasome and via autophagic pathway [80]. Consistently, autophagy suppression was associated with the generation of detergent-insoluble or high-molecular-weight oligomeric α-synuclein conformations, suggesting that autophagy dysfunction may lead to α-synuclein-related pathologies [81].

Taken together, our results provide evidence for a potential therapeutic role of BET inhibitors in counteracting the parkinsonian phenotype. PD is caused by a complex interplay of genetic and environmental factors affecting diverse molecular pathways that contribute to the degeneration of dopaminergic neurons [82]. Although extensive evidence supports the protective role of autophagy modulators and antioxidants in PD, alterations in these pathways do not act as sole drivers of pathology. This complexity suggests that effective therapeutic strategies should modulate multiple pathogenic mechanisms rather than targeting a single pathway [83,84]. In this context, the use of epigenetic modifying drugs, such as BET inhibitors, holds promise for their pleiotropic effects on the disease. Our work highlights that BET inhibition regulates redox metabolism and autophagy, along with mitochondrial homeostasis. Additional protective mechanisms triggered by BET modulation could be further explored.

Unlike current therapies, which primarily alleviate symptoms by compensating for reduced dopaminergic neurotransmission, BET inhibitors may offer a disease-modifying strategy by targeting cellular processes disrupted in Parkinson’s disease, thereby potentially enhancing the resilience of dopaminergic neurons. JQ1 derivatives, such as OTX-015, successfully entered clinical trials for other disorders, and demonstrated to have a good safety profile [85], suggesting that pharmacological blockade of BET proteins may be considered as an interesting therapeutic avenue. Nevertheless, further studies are needed to confirm the therapeutic potential of BET inhibition in PD, shedding light on the molecular mechanisms induced by BET inhibitors. In addition, the analysis of the potential effects of BET inhibitors should be extended to more complex in vivo experimental models. Indeed, although rotenone-based cell models are widely accepted to study the efficacy of pharmacological therapies in PD [86], the results need to be validated in animal models that may be useful to better assess dopaminergic neuronal cell survival, behavioral, and other functional manifestations related to PD pathophysiology.

## Data Availability

The data that support the findings of this study are available from the corresponding author upon reasonable request.

## Supplementary Materials

The following supporting information can be downloaded at: https://www.mdpi.com/article/10.3390/biomedicines14010244/s1. Figure S1: Evaluation of neuronal differentiation efficiency in SH-SY5Y cells. Figure S2: Quantitative analysis of oxidative damage markers. Figure S3: Evaluation of mitochondrial population. Figure S4: Quantitative analysis of transcription factors involved in redox metabolism and mitochondrial homeostasis. Figure S5: Impact of BET inhibition on the main pro-oxidant cell systems. Figure S6: Expression of autophagy-related proteins in SH-SY5Y cells and BET proteins in N1E-115 cells. Table S1: List of antibodies used in this study.
