# Supplementary material for: Modulation of Bromo- and Extra-Terminal Domain (BET) Proteins Exerts Neuroprotective Effects in Cell Culture Models of Parkinson’s Disease
Source: Biomedicines. 2026 Jan 21;14(1):244. doi: 10.3390/biomedicines14010244 (PMC12839369; doi:10.3390/biomedicines14010244)
Supplement: Supplementary file 1 [file biomedicines-14-00244-s001.zip › Supplementary figures_Martella et al. 2026_revised.pdf]

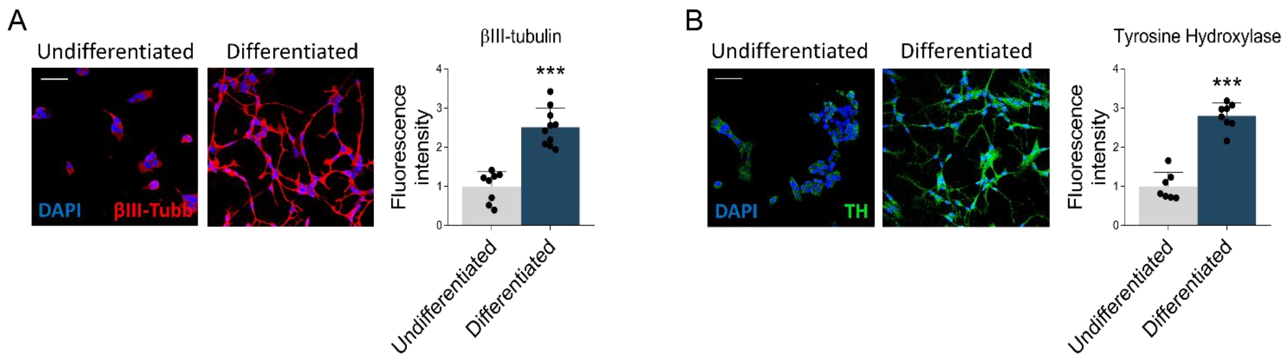

Figure S1. Evaluation of neuronal differentiation efficiency in SH-SY5Y cells. Confocal microscopy representative images and quantitative analysis of (A) βIII-tubulin (red) and (B) Tyrosine hydroxylase (green) in undifferentiated and differentiated SH-SY5Y cells. SH-SY5Y differentiation was induced in DMEM containing 1% FBS and 10 µM retinoic acid for 72 hours. DAPI (blue) was used to counterstain nuclei. N=7-10 independent experiments. Scale bar: 50 µm. Statistical analysis was performed by using the Unpaired Student's t-test. *** $p < 0.001$. Data are represented as means ± SD. The black dots depicted around the SD represent individual values, each derived from the average fluorescence calculated in the cells present in a single image (each image derives from a different experiment).

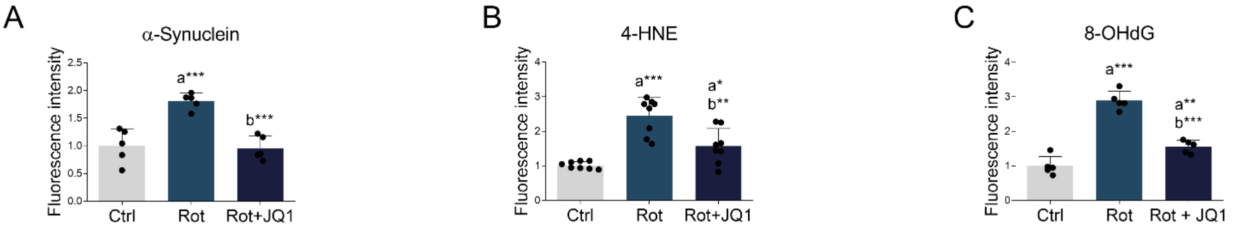

Figure S2. Quantitative analysis of oxidative damage markers. Immunofluorescence signal quantification of (A) α-synuclein, (B) 4-HNE, and (C) 8-OHdG in differentiated SH-SY5Y treated with DMSO (Ctrl), rotenone (Rot, 50 nM) or rotenone with JQ1 (Rot+JQ1, 100 nM) for 24h. N=5-8 independent experiments. Data are represented as means ± SD. Statistical analysis was assessed using one-way ANOVA, followed by Tukey's post hoc. "a" indicates statistical significance vs Ctrl; "b" indicates statistical significance vs rotenone group. * $p < 0.05$; ** $p < 0.01$; *** $p < 0.001$.

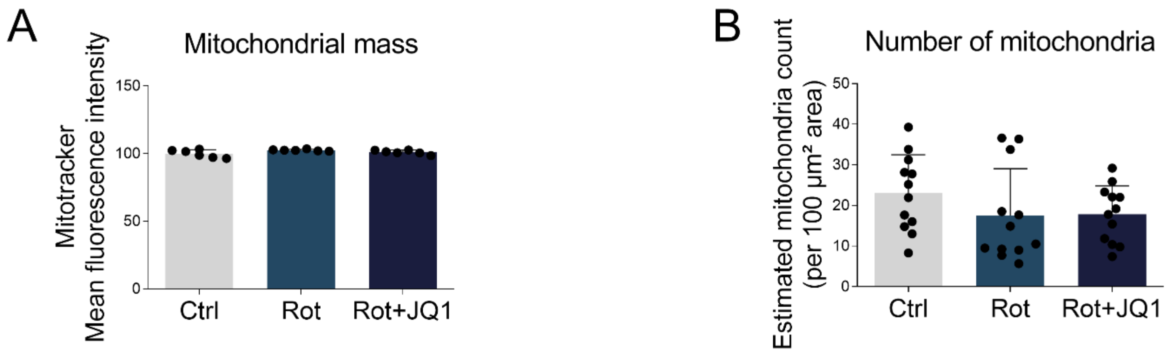

Figure S3. Evaluation of mitochondrial population. (A) Quantitative analysis of mitochondrial mass was assessed by estimating the fluorescence intensity of Mitotracker dye in differentiated SH-SY5Y treated with DMSO (Ctrl), rotenone (Rot, 50 nM) or rotenone with JQ1 (Rot+JQ1, 100 nM) for 24h. Mean fluorescence intensity was calculated by dividing total fluorescence by cell count. N=6 independent experiments. (B) Statistical analysis of mitochondrial number, quantified from FIB/SEM micrographs of the central region of the cell body in differentiated SH-SY5Y cells treated as in A). Counts were performed at varying magnifications and normalized to the number of mitochondria per 100 μm^2. Data are represented as means ± SD. Statistical analysis was assessed using one-way ANOVA, followed by Tukey's post hoc.

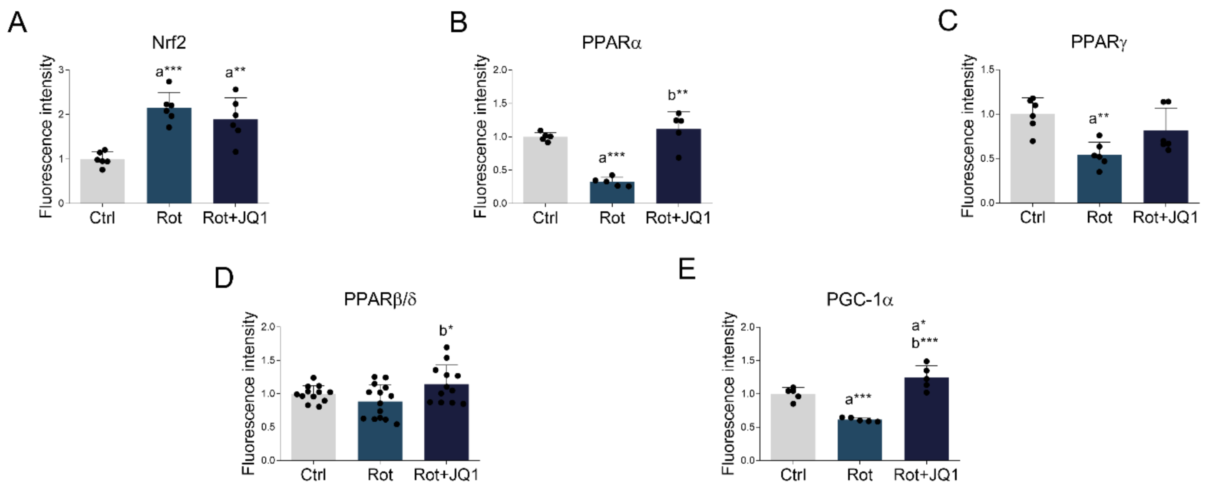

Figure S4. Quantitative analysis of transcription factors involved in redox metabolism and mitochondrial homeostasis. Immunofluorescence signal quantification of (A) Nrf2, (B) PPARα, (C) PPARγ, (D) PPARβ/δ, and (E) PGC-1α in differentiated SH-SY5Y treated with DMSO (Ctrl), rotenone (Rot, 50 nM) or rotenone with JQ1 (Rot+JQ1, 100 nM) for 24h. N=5-12 independent experiments. Data are represented as means ± SD. Statistical analysis was assessed using one-way ANOVA, followed by Tukey's post hoc. "a" indicates statistical significance vs Ctrl; "b" indicates statistical significance vs rotenone group. * $p < 0.05$; ** $p < 0.01$; *** $p < 0.001$.

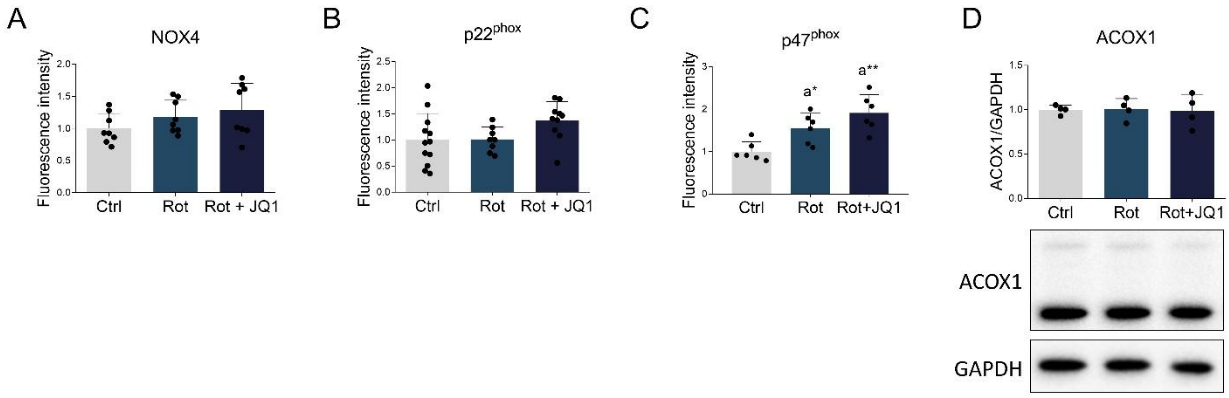

Figure S5. Impact of BET inhibition on the main pro-oxidant cell systems. Fluorescence intensity quantification of (A) NOX4, (B) p22phox, and (C) p47phox NADPH oxidase subunits in SH-SY5Y treated DMSO (Ctrl), rotenone (Rot, 50 nM) or rotenone with JQ1 (Rot+JQ1, 100 nM) for 24h. (D) Representative Western blot and densitometric analysis of ACOX1 in differentiated SH-SY5Y cells treated as indicated above. GAPDH was chosen as the loading control. Data are represented as means ± SD. N=4 experimental replicates. Statistical analysis was performed by using One-Way ANOVA followed by Tukey's post hoc test. "a" indicates statistical significance vs Ctrl. * *$p < 0.05$; *** *$p < 0.01$.*

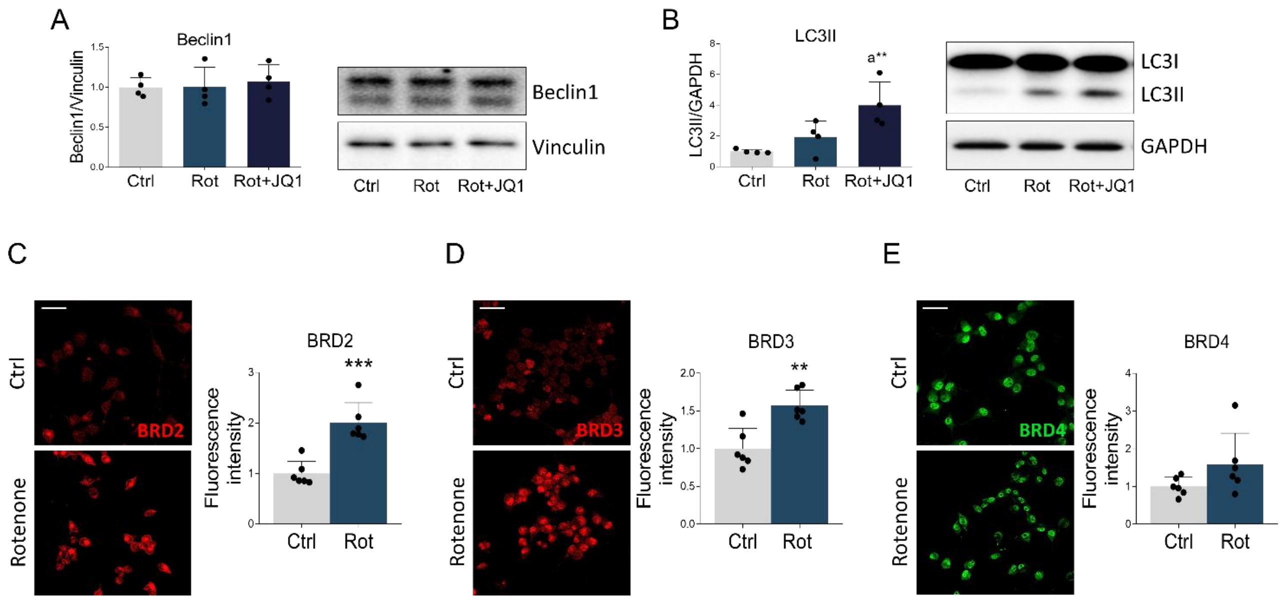

Figure S6. Expression of autophagy-related proteins in SH-SY5Y cells and BET proteins in N1E-115 cells. Representative immunoblot and densitometric analysis of (A) Beclin1 and (B) LC3 in lysates from differentiated SH-SY5Y treated with DMSO (Ctrl), rotenone (Rot, 50 nM) or rotenone with JQ1 (Rot+JQ1, 100 nM) for 24h. N=4 biological replicates. Vinculin and GAPDH served to normalize protein loading. Statistical analysis was performed by using One-Way ANOVA followed by Tukey's post hoc test. "a" indicates statistical significance vs Ctrl. ** *p < 0.01*. Confocal microscopy representative images and quantitative analysis of (C) BRD2 (red), (D) BRD3 (red), and (E) BRD4 (green) in differentiated N1E-115 cells treated with DMSO (Ctrl) and rotenone (Rot, 50 nM) for 24h. DAPI (blue) was used to counterstain nuclei. N=6 independent experiments. Scale bar: 50 µm. Statistical analysis was performed by using the Unpaired Student's t-test. ** *p < 0.01;* *** *p < 0.001*. Data are represented as means ± SD. The black dots depicted around the SD represent individual values, each derived from the average fluorescence calculated in the cells present in a single image (each image derives from a different experiment).
