# Supplementary material for: Modulation of Bromo- and Extra-Terminal Domain (BET) Proteins Exerts Neuroprotective Effects in Cell Culture Models of Parkinson’s Disease
Source: Biomedicines. 2026 Jan 21;14(1):244. doi: 10.3390/biomedicines14010244 (PMC12839369; doi:10.3390/biomedicines14010244)
Supplement: Supplementary file 1 [file biomedicines-14-00244-s001.zip › Supplementary table 1_Martella et al. 2026_revised.pdf]

Supplementary Table 1: List of antibodies used in this study

Antibody	Ref	Provider	Application and Dilution
4-HNE	MA527570	ThermoFisher Scientific	IF (1:100)
8-OHdG	sc-66036	Santa Cruz Biotechnology	IF (1:100)
βIII-tubulin	sc-80005	Santa Cruz Biotechnology	IF (1:200)
ACOX1	Ab184032	Abcam	WB (1:1000)
Akt1/2/3	sc-8312	Santa Cruz Biotechnology	WB (1:1000)
AMPK	sc-25792	Santa Cruz Biotechnology	WB (1:1000)
Beclin 1	sc-11427	Santa Cruz Biotechnology	WB (1:1000)
BRD2	sc-393720	Santa Cruz Biotechnology	IF (1:50)
BRD3	sc-81202	Santa Cruz Biotechnology	IF (1:50)
BRD4	NBP1-18874	Novus Biological	IF (1:500)
Catalase	sc-271803	Santa Cruz Biotechnology	WB (1:500)
GAPDH	sc-32233	Santa Cruz Biotechnology	WB (1:5000)
GPX1	Ab22604	Abcam	WB (1:1000)
GSH	Ab19534	Abcam	IF (1:150)
LC3	L7543	Merck Life Science	IF (1:400);<br>WB (1:2000)
NOX2	sc-130543	Santa Cruz Biotechnology	WB (1:1000)
NOX4	sc-518092	Santa Cruz Biotechnology	IF (1:50)
Nrf2	sc-365949	Santa Cruz Biotechnology	IF (1:100)
p22phox	sc-130551	Santa Cruz Biotechnology	IF (1:50)
p47phox	sc-17844	Santa Cruz Biotechnology	IF (1:50)
p-Akt	sc-7985	Santa Cruz Biotechnology	WB (1:500)
p-AMPK	sc-33524	Santa Cruz Biotechnology	WB (1:300)
PGC1α	sc-13067	Santa Cruz Biotechnology	IF (1:50)
PPARα	Ab8934	Abcam	IF (1:300)
PPARβ/δ	Ab32673	Abcam	IF (1:100)
PPARγ	Ab59256	Abcam	IF (1:100)
Sir2	#07-131	Upstate Cell Signaling Solutions	WB (1:500)
SOD1	sc-271014	Santa Cruz Biotechnology	WB (1:300)
SOD2	sc-137254	Santa Cruz Biotechnology	WB (1:300)

Tyrosine hydroxylase	701949	ThermoFisher Scientific	IF (1:100)
Vinculin	sc-73614	Santa Cruz Biotechnology	WB (1:500)
α-synuclein	sc-12767	Santa Cruz Biotechnology	IF (1:50)
Goat Anti-Mouse IgG (H + L)-HRP Conjugate	#1706516	Biorad Laboratories	WB (1:10000)
Goat Anti-Rabbit IgG (H + L)-HRP Conjugate	#1706515	Biorad Laboratories	WB (1:10000)
Goat anti-Rabbit IgG (Heavy chain), Superclonal™ Recombinant Secondary Antibody, Alexa Fluor™ 488	A27034	Thermo Fisher Scientific	IF (1:300)
Goat anti-Mouse IgG (H+L), Superclonal™ Recombinant Secondary Antibody, Alexa Fluor™ 555	A28180	Thermo Fisher Scientific	IF (1:300)
